# Patients Experience of Ear Acupuncture for Anxiety—A Qualitative Interview Study

**DOI:** 10.1155/nrp/5882178

**Published:** 2026-09-15

**Authors:** Anette Berggren, Roger Stjernkvist, Linda Hedberg, Caroline Larsson, Katarina Rusch, Harald Aiff

**Affiliations:** ^1^ Psychiatric Department of Halland, Region Halland, Varberg, Sweden; ^2^ Institute of Neuroscience and Physiology, University of Gothenburg, Gothenburg, Sweden, gu.se

## Abstract

**Background:**

This study aimed to qualitatively explore and understand the experiences of patients with anxiety undergoing National Acupuncture Detoxification Association (NADA) treatment in a psychiatric outpatient setting.

**Methods:**

Through interviews, 20 participants shared their experiences after receiving 10 NADA treatments. The data were meticulously analyzed using latent content analysis to identify recurring themes and patterns.

**Results:**

Four themes emerged from the analysis: “A gradually increasing sense of well‐being,” “Relief that lasted temporarily or throughout the treatment period,” “A feeling of inner peace and relaxation,” and “A transient feeling of discomfort and disappointment,” reflecting ambivalence regarding the tangible benefits of the treatment.

**Conclusion:**

The findings reveal a multifaceted landscape of experiences with NADA treatment for anxiety. While the majority experienced relief and anticipatory sentiments, it was evident that the benefits were not universally experienced. The study underscores the need for continued research and optimization of the NADA protocol to cater to diverse patient needs within psychiatric outpatient settings.

**Relevance to Clinical Practice:**

NADA treatment could pose a nonpharmacological option for some patients with anxiety. Patients experienced anxiety relief, and many wanted to continue treatment.

**Patient or Public Contribution:**

Patients/users of outpatient psychiatric care participated in the study as participants. They were then interviewed to share their experience.

**Summary**
• What does this paper contribute to the wider global clinical community?
◦ Some patients with anxiety might benefit from NADA treatment
◦ Some patients experienced relief and wanted to continue treatment

## 1. Background

Anxiety is a globally prevalent mental health concern highlighted by the World Health Organization (WHO). With a spectrum ranging from mild discomfort to severe distress and suicidal ideation, anxiety significantly impacts one’s quality of life [1]. Given its recurrent or chronic nature, it remains a predominant reason for medical consultation [2]. Despite the availability of various treatments such as psychological and pharmacological methods, only 10% of those diagnosed with anxiety receive adequate treatment [3]. The interest in acupuncture as a nonpharmacological treatment method has increased, and acupuncture is used to some extent as an adjunct or complement to traditional treatment in psychiatric care [4, 5]. Acupuncture may also serve as an alternative to traditional psychotherapy, particularly for patients who face barriers related to language, verbal expression, or the interpersonal demands of talk therapy.

Acupuncture has been an integral part of Asian medical practices for thousands of years [6]. In its various methodologies [7], one noteworthy approach is the National Acupuncture Detoxification Association (NADA) protocol conceptualized by Dr. Michael Smith in the 1970s [8]. Predominantly recognized for its use in substance use and psychiatric care in the United States, Germany, Italy, and Sweden, NADA distinguishes itself through a standardized protocol including five specific ear acupuncture points [9].

Several anecdotal and empirical insights hint at the NADA treatment’s potential in alleviating anxiety [8]. Furthermore, the incorporation of acupuncture in therapeutic settings has been reported to empower and validate the roles of psychiatric caregivers [10]. In one qualitative analysis of patients with eating disorders treated with NADA, patients experienced anxiety relief [11]. Ear acupuncture seems to have similar effects to muscle relaxation techniques, suggesting that it may be a treatment alternative in some cases [12, 13]. Acupuncture was a good treatment option to reduce patient anxiety in a primary care setting, but [14] concluded that more research is needed to confirm the study’s results. A systematic review and meta‐analysis of ear acupuncture in anxiety disorders has shown the intervention may reduce anxiety levels compared to waiting list and placebo with moderate certainty [15]. Auricular acupuncture may reduce anxiety by stimulating the auricular branch of the vagus nerve, enhancing vagal afferent signaling to the brainstem and promoting parasympathetic activity. This mechanism is supported by evidence from transcutaneous auricular vagus nerve stimulation studies showing reduced anxiety and improved autonomic regulation [16].

Based on clinical experience with acupuncture treatment among professionals in the clinic, we chose to add three more needles to the NADA protocol, one in the scalp (GV20) and one in each little finger (HT9). This was believed to enhance the effect of the NADA treatment, although this hypothesis has not been tested yet.

Patients with anxiety having contact with psychiatric outpatient care have, in many cases, tried multiple treatments for their anxiety with insufficient relief. We wanted to investigate if the enhanced NADA protocol could be a viable option as an adjunct to usual care for these patients. This study, therefore, seeks to delve deeper into the lived experiences of patients undergoing NADA treatment for anxiety symptoms.

## 2. Methods

This study used latent content analysis to identify recurring themes and patterns in interviews with patients with anxiety symptoms who underwent NADA treatment combined with body acupuncture. The insights are primarily based on personal narratives captured through participant interviews.

### 2.1. Study Approval and Ethics

Conducted between September 2019 and June 2020, this study was sanctioned by the Swedish Ethical Review Authority (No. 2019‐03292) and adheres to the ethical principles outlined in the Helsinki Declaration.

### 2.2. Participants and Recruitment

Eligible participants were adult patients (≥ 18 years) self‐reporting experiencing anxiety, attending outpatient psychiatric care in northern Halland County, Sweden. Healthcare professionals introduced the study verbally and through written documents. Interested patients were briefed by the study coordinator, after which written consent was collected from the patients who agreed to participate.

All patients continued their usual psychiatric care during the study, including psychological and medical treatment. NADA was not part of the standard care in outpatient psychiatric services before or during the study period.

Patients with conditions such as psychotic disorders, active substance use, severe suicidality, unstable bipolar disorder, or any other severe affective disorders were not included in the study.

A total of 27 patients were enrolled in the study. Seven participants discontinued participation before completing all treatments and did not take part in the interview. Recruitment concluded when 20 participants (4 men and 16 women) had completed all 10 NADA sessions.

### 2.3. Treatment Procedure

**FIGURE 1:**
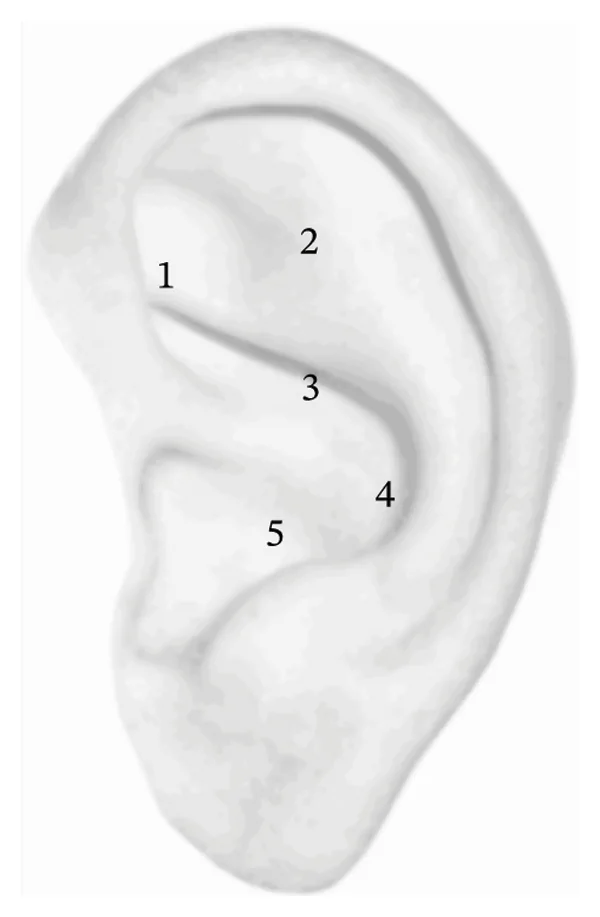
NADA treatment points. 1: Sympathetic; 2: shen men; 3: kidney; 4: liver; 5: lung.

The participants received the NADA treatment at one of the outpatient care facilities in northern Halland County, Sweden, in which three nurses were NADA‐trained. The treatment was provided with the patient sitting in a room specifically set aside for the NADA treatment. The room was quiet and without other stimuli. After disinfection of the skin, 13 needles (Phoenix A‐type Acupuncture needles, size: 0.20 × 13 mm) were inserted by the nurse in accordance with the NADA treatment protocol: five into each ear (Figure 1), together with one in the middle of the scalp (GV20) and one in each little finger (HT9). The participants were then left alone for 40 min before the nurse removed the needles and the participants returned home. Participants received treatment twice a week for 5 weeks, thus a total of 10 treatment sessions.

### 2.4. Data Collection

Participants were interviewed after the 10 treatment sessions. The interview began with an open‐ended question, “Can you please describe your experience with NADA treatment?” Participants were encouraged to freely express their feelings, experiences, and perceptions. Probing questions were sparingly used when considered necessary to cover all aspects of the treatment experience. Interviews were conducted by nurses who did not take part in the treatment procedure. This was done to ensure that participants could express themselves freely. Each interview was digitally captured, except for one lost recording. The duration ranged from 20 min to 1.5 h, depending on the individual patient’s willingness to elaborate and provide explanations. The interviews were performed within 1‐2 weeks after the last NADA treatment.

Participants also answered self‐report questionnaires for a quantitative study which will be reported elsewhere.

### 2.5. Data Analysis

The data analysis was performed by AB, RS, and LH. The interviews were initially listened to several times with an open approach. The material from the recordings was then transcribed verbatim. To get a summarizing impression and to become familiar with the material, the transcribed material was read several times in accordance with [17]. Meaning‐bearing parts of the text, so‐called meaning units, which can be sentences or words, form the basis for the analysis [18]. In each interview, the meaning units that answered the purpose of the study were extracted, condensed, and then coded as described by [17]. The coding was done with latent content analysis, whereby the text was abstracted and the content elevated to a higher level. The analysis continued by abstracting the content of the codes into subthemes and finally themes. Several codes with similar content formed a subtheme, and by combining the underlying messages, themes were formed [18]. A coding scheme was created to organize data according to content in different codes and subthemes. The categorization of the content meant that data that did not respond to the purpose were sorted out. A theme answered the question “What?” and data could only fit into one theme, but a theme could be divided into two or more subthemes [17] (see Table 1).

**TABLE 1:** Examples of coding from meaning‐bearing unit to theme.

Meaning‐bearing unit	Condensed unit	Code	Subtheme	Theme
1. Eh, yes, it was; it’s probably the sleep. But it was also like that maybe 2‐3 nights after. Because then I got it again.	The sleep got better and lasted 2‐3 nights	Better sleep	A feeling of relaxation and a pleasant increase in tiredness that contributes to better sleep quality	A gradually increasing sense of well‐being
1. Yeah… I didn’t wake up with anxiety either, and I usually do, but I didn’t now either. Nah.	I didn’t wake up with anxiety like I usually do	Wake up without anxiety	A feeling of serenity and inner peace	
2. It was on several occasions that I really felt relaxation. Others not so much. But that may well be the case.	Felt relaxed on several occasions, but the degree of relaxation varied.	Various degrees of relaxation	A pleasant physical rest	
8. I think I’ve calmed down and become more harmonious as well… Not so high and low all the time like it was for a while.	I am calmer and more harmonious, and I am less up and down	More calm and stable	A sense of calm and harmony	
1. “Uh, nah, and what I mainly felt was that, I have a lot of heart palpitations and stuff and it got a lot a lot better.”	Problem with heart palpitations became much better	Less heart palpitations	Relief of bodily discomfort	
14. “I feel like I’m going in pretty much set to zero, then notice that the phase helps and it’s good.”	I notice that it helps and that it is “good”	Feels good	It helps and does good	

## 3. Results

Four men and 16 women were interviewed. The average age was 42 years.

The analysis of the collected material resulted in four themes: “A gradually increasing sense of well‐being,” “Relief that lasted temporarily or throughout the treatment period,” “A feeling of inner peace and relaxation,” and “A transient feeling of discomfort and disappointment.” The themes revealed how participants with anxiety in psychiatric outpatient care experienced NADA treatment.

### 3.1. A Gradually Increasing Sense of Well‐Being

The participants experienced the treatment as enjoyable and felt that it improved over time. The effect came gradually and increased with the number of sessions. Several participants stated that a couple of treatment sessions were needed before well‐being gradually increased and an inner peace emerged. Some participants found it difficult to relax and slowdown in connection with the treatment, something they describe as becoming easier over time. Some participants described looking forward to the treatment, as it gave them a good feeling. One participant described this as:

“The very first time I had a bit of a hard time relaxing. And I did not feel that it gave me much. The second time it felt a little better but then after round three I really felt that it worked for me…. I kind of longed for every… for every occasion… Yes, it felt like you had been to a really nice massage” (P1).

Participants described NADA treatment as an occasion to look forward to and yearn for, and that instills hope. NADA treatment was also described as helping them fall asleep, and for some, expressed hopes of being able to stop taking their medications. One participant described a longing for the NADA treatment despite the participant not liking needles:

“that I actually longed to receive such a treatment at the same time that I didn′t like it, I don′t like needles” (P5)

### 3.2. Relief That Lasted Temporarily or Throughout the Treatment Period

Some participants experienced immediate relief after the first treatment session, which lasted throughout the entire treatment period. Others needed a few treatments before they experienced relief. The relief also persisted between treatment sessions, but the anxiety returned a couple of days to weeks after the treatment period had ended. Some participants felt that the continuity between treatments was good, as the experienced effect of the NADA treatment did not diminish between treatments.

One participant experienced that the relief lasted a few weeks after the end of the NADA treatment:

“During the treatment itself I had less anxiety and it was easier. But then after a week or so it got worse. It really helped while I was receiving the treatment.” (P20).

Another participant said that her husband noticed a difference in her mood after she finished the NADA treatment:

“And I noticed it even more when I stopped it. Yes, the anxiety rose and it was like it was actually my husband who said it “but now you have stopped the acupuncture!” (P2)

Some participants experienced relief immediately following the treatment, which could vary from an hour up to a day. The relief varied from person to person but could also vary from one occasion to another. Between treatment sessions, however, anxiety returned. Although the relief was temporary, it was perceived as positive to feel relief in the moment.

Some participants described that with the help of NADA treatment, they were able to achieve relief a few days a week, something that one patient described as:

“What I noticed, on the days I didn′t have the acupuncture, I became a little more anxious. I noticed it, I could see it.” (P8)

### 3.3. A Feeling of Inner Peace and Relaxation

Several participants felt they relaxed after the NADA treatment. One participant described it as a nice massage and stated a feeling of harmony. Other participants described a better feeling in the body, that the body relaxed, and meant that the participant could fall asleep. One participant experienced the NADA treatment as the calmest moment in a long time and stated that the calming effect lasted even a few hours after the NADA treatment. One participant stated:

“Because I became very tired and relaxed, so I think it means a lot to me, to feel relaxed in my body, which I haven′t felt in years.” (P4)

One participant felt that the NADA treatment brought the participant closer to the feelings and being more “in phase.” Another felt that the calm the treatment brought helped with the negative thoughts. Several participants stated that the NADA treatment resulted in relaxation, which sometimes resulted in the participant falling asleep during the NADA treatment, but also that falling asleep between NADA treatments was facilitated.

Participants reported that it led to improved sleep quality, increased relaxation, and a growing feeling of fatigue, which facilitated sleep. Several participants experienced a significant improvement in sleep from the NADA treatment. Some participants had improved sleep the night after treatment, while other participants experienced improved sleep for several nights. Some participants also experienced falling asleep faster. One participant wished to learn to give himself NADA acupuncture every night because sleep worked so well from the treatment. Another participant described that sleep improved so much that the participant might be able to stop his sleep medications if the treatment continued:

“It was much easier for me to fall asleep because I became so tired. It helped me along the way to maybe being able to try to manage without my sleeping pills.” (P10).

“I take sleep medication and what if this would mean that I won’t have to take any medicine?” (P5)

NADA treatment reduced perceived anxiety in several of the participants. The participants experienced anxiety relief in direct connection to the NADA treatment, but also relief that lasted for a longer period of time, ranging from hours after the treatment to a few days after. Several participants also described reducing their anti‐anxiety medication while receiving NADA treatment. The participants also described that the anxiety they experienced became easier to handle and that anxiety attacks subsided more quickly than usual. The time spent thinking about the anxiety also decreased during the treatment period. One participant described it as:

“I thought that during the treatment itself, I thought I had less anxiety and it went easier. But then a week or so later it got worse, and I noticed it even more when I stopped it. Yes, the anxiety rose, so it feels like it really helped during the time I got it” (P20).

Several participants felt that the NADA treatment gave them a relaxed feeling in the body and also a greater sense of calm. The results show that the relaxation often lasted throughout the day, but that the degree of relaxation could vary from time to time. One participant felt that the NADA treatment provided better relaxation than the medications:

“And I haven′t felt like even with medication that I′ve been able to become the way I′ve become with this, so it′s been a huge saving from that.” (P4)

Several participants experienced a feeling of calm and harmony in connection with the NADA treatment. One participant described feeling anxious during the first treatments, but that this anxiety was then replaced by an inner calm. Other participants described an inner calm that was evident already after the first treatment. The relaxation made possible by NADA was appreciated by the participants. One participant described an experience of calm and harmony and also a feeling of a more stable state of mind:

“I think I′ve calmed down and become more harmonious as well. And I noticed it even more when I stopped it. Yes, the anxiety rose not as high and low all the time as it was for a while.” (P8)

Some participants reported relief of physical symptoms such as palpitations and sweating associated with anxiety attacks:

“Uh, nah, and what I mainly felt was that, I have a lot of heart palpitations and stuff and it got a lot a lot better.” (P1)

The result shows that NADA treatment is a treatment that is appreciated among the participants. Several participants wished to continue the treatment after the end of the study as they felt helped by the NADA treatment. The participants described the NADA treatment as a treatment that really worked, and that does good:

“I feel like I′m going in pretty much set to zero, then notice that the phase helps and it′s good.” (P14).

### 3.4. A Transient Feeling of Discomfort and Disappointment

The results showed that several participants experienced physical discomfort in the form of, among other things, pain and dizziness. The pain caused tension and difficulty relaxing, sometimes even bleeding. Several participants had headaches that persisted in connection with the NADA treatment, but in some cases also for a while after the treatment. Some participants experienced dizziness during the first treatment, and some experienced dizziness at the beginning of each treatment. The insertion of the needles could be uncomfortable or painful, especially in the fingers, which one participant described as:

“It was just when she put them in but then it could… in the little finger so it could ache in the whole hand. Sometimes it hurt my ear.” (P18).

One participant described the NADA treatment as painful, but she endured the treatment for the pain‐relieving and calming effect, described as:

“the fingers hurt and it really tensed up sometimes but I often persevered because it started a pain‐relieving and calming system” (P5)

The result showed that some participants had difficulty relaxing during the NADA treatment. Sometimes because of fear of pain or that it was quiet in the treatment room, but also because of fear of losing control, which one participant described as:

“Then I almost got scared or I was scared, and I think it is very uncomfortable because I feel like I am losing control as well.” (P5)

The results also show that some participants felt that it was difficult to get to the treatment, which made it difficult for the participant to follow the treatment:

“I want to do it because it feels good, but for me, it becomes a big project because I have to go out even though I really just want to stay inside. If I had a choice, maybe I would take some medicine instead because then I don′t need to go outside. But perhaps it′s good to be pushed.” (P 10)

Some participants experienced a lack of—or were doubtful about—the benefit of the treatment, which was described as a doubt as to whether the anxiety had decreased or if they felt more relaxed in connection with NADA. One participant was doubtful about whether it brought any relief from anxiety but did not report any negative experiences:

“But if it reduced my anxiety, I′m very uncertain. It′s hard to say if I was more relaxed afterwards … nothing got worse in any way. I did not experience any negative effects. But I don’t know whether it made the situation any easier” (P6).

## 4. Discussion

Our study indicates that some participants perceived NADA as an effective treatment. Some participants even argued that NADA was the most effective treatment they had ever tried. It often takes multiple treatment sessions before any perceived effect is seen; hence, the treatment is not a “quick fix” for anxiety. Many participants, even those who did not experience any effect, were grateful for the opportunity to try a nonpharmacological treatment. NADA treatment was perceived to alleviate the participants’ suffering, giving them hope that they would be able to reduce or stop their medication in the future.

The study also indicated that NADA treatment may offer benefits beyond anxiety relief. Participants reported improvements in sleep quality, reductions in heart palpitations and sweating, as well as enhanced overall well‐being and a greater sense of harmony. Given that anxiety commonly manifests through symptoms such as sleep disturbances, excessive sweating, and palpitations—and negatively impacts general well‐being—it is possible that these reported improvements reflect a reduction in anxiety‐related symptoms. Rather than explicitly identifying a decrease in anxiety per se, participants may have described relief from the physical and emotional symptoms associated with their anxiety.

Using NADA in participants in psychiatric settings has been studied previously. Participants experienced decreased cravings, pain, anxiety, stress, and depression [19] and increased relaxation and improved focus and concentration [20]. However, it has not been shown to be effective in reducing anxiety in patients withdrawing from psychoactive drugs [21].

Anxiety symptoms are more prevalent in women, which is also reflected in our study. However, as most of the study participants were women, it is not possible to state with certainty that women’s and men’s experience of NADA treatment for anxiety is the same. In one earlier study involving women, an anxiety‐alleviating effect was demonstrated [15].

All participants who started the study did not complete the study for various reasons. Just as with pharmacological treatment and other nonpharmacological treatments such as therapy, it seems of great importance that the patient who is offered NADA treatment is motivated to complete the treatment. The participants should also be informed that the treatment involves needles, needs to take place at a certain time interval, and where the treatment is carried out before the treatment. This is to make it easier for the patient to complete the entire treatment.

In most treatments, a motivated patient is more compliant, which is also true of NADA treatment. It is therefore important that the patient who is offered NADA treatment is not too afraid or finds the treatment procedure too painful, as this can lessen her/his compliance. Some participants experience pain during insertion of the needles. In our study, some of the participants who experienced pain still wanted to continue treatment due to the perceived positive effect on anxiety. Patients with anxiety are often tense and have difficulties relaxing. Some of our participants experienced this in the treatment situation, which could have diminished the treatment effect and the result of the study.

The study demonstrates that the perceived effect of NADA treatment varies among participants, as shown in a study by [22]. In some participants, it had a perceived positive effect on anxiety, but not in others. Some also noticed a positive impact on sleep, pain, and feeling tense. Many participants felt tired and relaxed during the treatment period. We interpret this as an indirect sign of anxiety relief. These perceived benefits are also shown in a study by [14], where body acupuncture had a strong anxiety‐relieving effect.

For some participants, the perceived effect of NADA lasted longer (days) than a typical anxiolytic medication (hours). Some participants also stated that the need for medication “as‐needed” was decreased with ongoing NADA treatment. This observation suggests that NADA could be a potential alternative method to decrease use of anxiolytics, that has multiple side effects (i.e., sedation, cognitive impairment, dependence).

Some participants became sleepy because of the NADA treatment and therefore suggested that it would be preferable for them to receive the treatment at home, just before bedtime. A few stated that they could have stopped their sleep medication if they could have continued the NADA treatment. This observation is supported in a study by [4], where it was shown that NADA treatment reduced the need for sleep medication. For these participants, as well as those who find it difficult to come to the clinic due to anxiety, it would be preferable if NADA treatment was available at home. In our study, the NADA treatment was administered at a psychiatric outpatient clinic. However, based on the findings of this study, we suggest offering NADA treatment at home either by an educated NADA acupuncturist or by self‐administration. If patients were able to obtain the treatment on a regular basis, it might reduce the use of pharmacological treatment “as needed.” Self‐administration requires a compliant patient as well as comprehensive patient education.

### 4.1. Relevance to Clinical Practice

NADA treatment could pose a nonpharmacological option for some patients with anxiety. Patients with anxiety symptoms for whom pharmacological treatment is not working or have an unfavorable side effect profile may be interested in NADA treatment. The treatment is safe and relatively well tolerated.

## 5. Conclusions

The findings reveal a multifaceted landscape of participant experiences with NADA treatment for anxiety. While the majority experienced relief and anticipatory sentiments, it was evident that the benefits were not universally experienced. The study underscores the need for continued research and optimization of the NADA protocol to cater to diverse patient needs within psychiatric outpatient settings.

### 5.1. Limitations

The COVID‐19 pandemic started at the end of the NADA treatment, and it is difficult to know how this affected the participants’ experience. Due to the pandemic, two interviews could not be conducted in conjunction with the end of the treatment, which could have affected the participants’ memories of the NADA treatment. The result may also be affected by the fact that the interviews were conducted by several different persons.

Some of our participants had previous experience of NADA treatment, which could indicate a pre‐existing positive attitude toward the treatment. The fact that such a large proportion of the participants had previous experience of the treatment may also indicate that those surveyed who were skeptical or already had negative experiences of NADA treatment declined.

In this kind of study, there is always a risk that participants feel obliged to answer the questions in a way that they think the researcher would prefer. To reduce this risk, the NADA treatment and the interviews were conducted separately by different persons.

One limitation of the study is the attrition rate, as seven participants discontinued treatment, leaving only 20 who completed the full intervention. It is possible that those who dropped out did so because they experienced the treatment as ineffective or uncomfortable, which may have introduced a selection bias in the reported outcomes.

## Author Contributions

Harald Aiff, Linda Hedberg, Anette Berggren, and Roger Stjernkvist collected the data, did the analysis, and wrote the paper.

Caroline Larsson and Katarina Rusch performed the NADA treatments.

## Funding

The work was funded by the Psychiatric Department of Halland, Sweden.

## Disclosure

All authors approved the final version.

## Ethics Statement

The study was approved by the Swedish Ethical Review Authority no. 2019‐03292 and conformed to the Declaration of Helsinki.

## Consent

All patients consented in writing to participate in the study.

## Conflicts of Interest

The authors declare no conflicts of interest.

## Data Availability

The data that support the findings of this study are available on request from the corresponding author. The data are not publicly available due to privacy or ethical restrictions.
